# A non-invasive risk score for predicting incident diabetes among rural Chinese people: A village-based cohort study

**DOI:** 10.1371/journal.pone.0186172

**Published:** 2017-11-02

**Authors:** Jiangping Wen, Jie Hao, Yuanbo Liang, Sizhen Li, Kai Cao, Xilin Lu, Xinxin Lu, Ningli Wang

**Affiliations:** 1 Department of Laboratory Medicine, Beijing Tongren Hospital, Capital Medical University, Beijing, China; 2 Beijing Tongren Eye Center, Beijing Tongren Hospital, Capital Medical University, Beijing Ophthalmology and Visual Science Key Laboratory, Beijing, China; 3 Clinical and Epidemiological Research Center, the Affiliated Eye Hospital of Wenzhou Medical University, Wenzhou, China; 4 Nanjing Aier Eye Hospital, Nanjing, China; 5 Beijing Institute of Ophthalmology, Beijing, China; 6 Department of Laboratory Medicine, Handan 3rd Hospital, Handan, China; Shanghai Diabetes Institute, CHINA

## Abstract

**Objective:**

To develop a new non-invasive risk score for predicting incident diabetes in a rural Chinese population.

**Methods:**

Data from the Handan Eye Study conducted from 2006–2013 were utilized as part of this analysis. The present study utilized data generated from 4132 participants who were ≥30 years of age. A non-invasive risk model was derived using two-thirds of the sample cohort (selected randomly) using stepwise logistic regression. The model was subsequently validated using data from individuals from the final third of the sample cohort. In addition, a simple point system for incident diabetes was generated according to the procedures described in the Framingham Study. Incident diabetes was defined as follows: (1) fasting plasma glucose (FPG) ≥ 7.0 mmol/L; or (2) hemoglobin A1c (HbA1c) ≥ 6.5%; or (3) self-reported diagnosis of diabetes or use of anti-diabetic medications during the follow-up period.

**Results:**

The simple non-invasive risk score included age (8 points), Body mass index (BMI) (3 points), waist circumference (WC) (7 points), and family history of diabetes (9 points). The score ranged from 0 to 27 and the area under the receiver operating curve (AUC) of the score was 0.686 in the validation sample. At the optimal cutoff value (which was 9), the sensitivity and specificity were 74.32% and 58.82%, respectively.

**Conclusions:**

Using information based upon age, BMI, WC, and family history of diabetes, we developed a simple new non-invasive risk score for predicting diabetes onset in a rural Chinese population, using information from individuals aged 30 years of age and older. The new risk score proved to be more optimal in the prediction of incident diabetes than most of the existing risk scores developed in Western and Asian countries. This score system will aid in the identification of individuals who are at risk of developing incident diabetes in rural China.

## Introduction

With the rapid development of the national economy and the changing lifestyle in China, the prevalence of diabetes and pre-diabetes has increased dramatically from 2.5% and 3.2%, in 1994 [1], respectively, to 11.6% and 50.1%, in 2010 [2], respectively. Thus, diabetes prevention has become a major public health issue in China. Urgent strategies are required to facilitate the prevention of diabetes in at-risk groups. Randomized controlled trials have shown that individuals at high risk of developing Type 2 diabetes (T2D) can significantly decrease the risk of diabetes onset following early interventions [3–7]. Therefore, appropriate identification methods that highlight individuals at high risk of developing incident diabetes are extremely important.

Several non-invasive risk scores for predicting incident diabetes have been developed and validated in western populations [8–13]. These non-invasive risk scores are based on non-laboratory clinical information and do not require blood tests. They have been suggested as useful tools in screening individuals at high risk of developing T2D in the general population [14], particularly in underdeveloped areas. However, the predictive performance of diabetes risk scores varies with country, age, sex, and adiposity [14], and therefore, an ethnic- or country-specific risk score is needed. To date, none of the reported non-invasive risk scores has been based on longitudinal cohort studies of the Chinese population, although two non-invasive risk scores based on cross-sectional surveys have been developed to detect undiagnosed diabetes in China [15, 16].

In China, approximately half of the population live in rural areas and compared with urban residents, their diet, education, economic income, and diabetes prevalence varies considerably [17]. Therefore, in this study, we report a simple non-invasive risk score for predicting incident diabetes using a village-based cohort of rural Chinese people aged 30 years and older, in Handan, Hebei province.

## Materials and methods

### Study population

We performed an analysis using longitudinal data from the Handan Eye Study (HES). Detailed information about the methods and procedures pertaining to this survey is available elsewhere [18]. The HES is a village-based cohort study designed to survey eye diseases and other health-related problems in non-institutionalized, community-dwelling persons, aged 30–97 years in Yongnian, which is a rural county of Handan and located approximately 500 km south of Beijing. The population was approximately 830,000 in 2000. In this region, 80% of the population engages in farming, and 98% are of Han ethnicity. Per capita net income of rural households in this region is 3,468 Yuan (approximately 468 USD), which is similar to the average income (3,587 Yuan, 484 USD) for the People's Republic of China [19]. This study was approved by the Ethics Committee of Beijing Tongren Hospital (approval number # TREC2006-22) and all study procedures adhered to recommendations of the Declaration of Helsinki. Written informed consent was obtained from all subjects. A stamp of the right forefinger was accepted as an alternative to a signature from those who could not read or write. This strategy was approved by the Ethics Committee.

**Fig 1:**
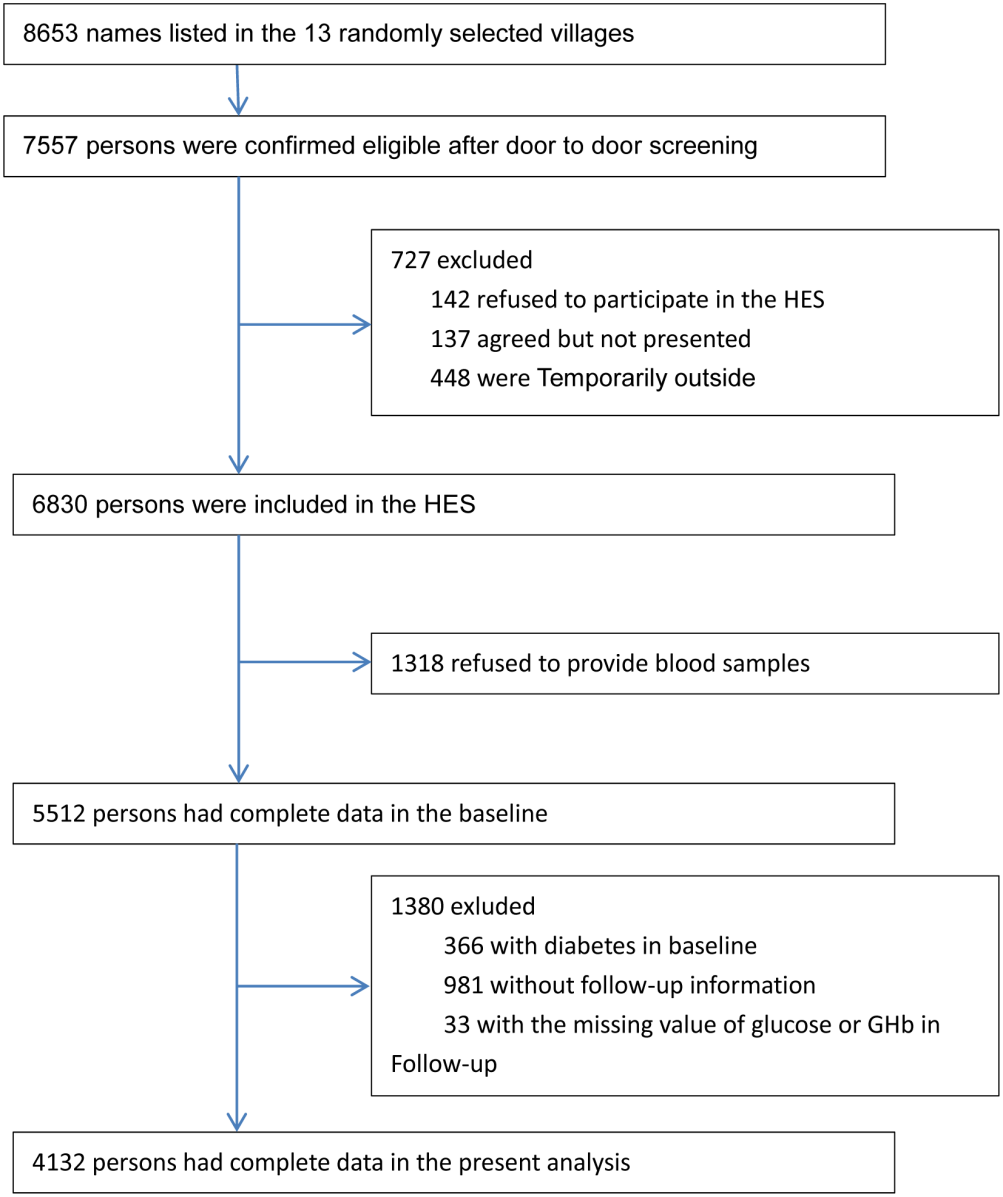
Flow diagram of recruitment of participants in the analysis.

Residents of Yongnian County, aged 30 years or older were randomly selected using a cluster sampling technique, with probabilities, proportional to the size (PPS) of the population in each cluster. Out of 453 villages, 13 villages in Yongnian County were selected to participate. Participants aged ≥50 years were selected from these 13 villages; however, a random selection was also made of those aged between 30–49 years in six of the 13 villages. As illustrated in Fig 1, of the 8,653 individuals screened for HES, 7,557 were found to be eligible. A total of 6,830 participants took part in the HES from October 2006 to October 2007, with a follow-up survey conducted between 2012 and 2013. We excluded 366 individuals who at baseline were diagnosed as diabetic. This determination was made following clinical diagnosis or upon observation that fasting plasma glucose (FPG) test results were ≥7.0 mmol/L (an oral glucose tolerance test or hemoglobin A1c (HbA1c) testing were not performed at baseline). A total of 1318 individuals refused to provide a blood sample at baseline, 981 individuals did not present with follow-up information, and 33 individuals were missing glucose or HbA1c test values during follow-up examination, thereby leaving 4,132 participants for the present analysis.

### Data collection

Trained interviewers used questionnaires to obtain answers pertaining to demographic information, including date of birth, gender, ethnicity, occupation, education, health status, health behavior (smoking, alcohol use and physical activity), medical history, and family history of diabetes (parents or siblings). Participant education was categorized into four groups according to the number of years of education (illiterate, 0 years; primary school, 1–6 years; junior high school, 7–9 years; and senior high school, ≥10 years). Physical activity was classified as low (exercising rarely or never), moderate (walking or bicycling continually for more than 10 min, 1–3 times/week), and high (exercise that causes rapid respiration for more than 10 min, >3 times/week).

During the clinical examination, two blood pressure measurements were obtained using a noninvasive, automated Hem-907 blood pressure monitor (OMRON, Japan). To conduct these measurements, participants were placed in a seated position after five minutes of rest. Systolic blood pressure (SBP) and diastolic blood pressure (DBP) were calculated as the mean of the two independent measurements. Hypertension was positively identified if the individual presented with SBP ≥140 mmHg, or DBP ≥90 mmHg, or if antihypertensive medication was used. Body height and weight were measured with subjects not wearing shoes or outerwear. Height measurements were performed with a wall-mounted measuring tape. Weight measurements were taken using a bathroom scale (RGZ-120, China). Body mass index (BMI) was calculated as weight (kg)/height (m^2^). The location of the waist measurement was set as the mid-point between the last rib and the iliac crest in the mid-axillary line.

Every participant in the study was requested to fast for at least 8 h prior to blood drawing, which took place between 7:00 and 9:00 a.m. in the villages. Blood samples were collected in a 3-ml vacuum tube containing sodium fluoride for blood glucose testing and a second vacuum tube containing no additives was used for other biochemical analyses. FPG was measured by a hexokinase method (Olympus AU2700, Japan). HbA1c was quantified by high-performance liquid chromatography (Bio-Rad D10, USA) and measurements were traceable to DCCT/NGSP.

We defined incident diabetes as follows: (1) FPG ≥7.0 mmol/L; or (2) HbA1c ≥6.5%; or (3) self-reported diagnosis of diabetes or use of anti-diabetic medications during the follow-up period.

### Statistical analysis

Statistical analysis was performed with SPSS (Statistical Package for the Social Sciences) v.18.0 software. The current analysis was restricted to 4,132 subjects who presented with complete diabetes data (S1 Dataset). The baseline characteristics of the participants were described separately for men and women, using means ± SD or median (interquartile range) for continuous variables, and counts and percentages for categorical variables. For comparisons of the mean or median values, the unpaired t-test or Mann-Whitney U test were used. Categorical variables were analyzed using a χ^2^ test.

Forward stepwise logistic regressions were used to investigate significant non-invasive risk factors for predicting incident diabetes in two-thirds of the sample cohort (these individuals were randomly chosen and this cohort was designated as the “training sample”). Candidate risk factors suggested by previous studies included age, sex, BMI, waist circumference, waist-to-hip ratio, SBP, DBP, smoking, alcohol use, family history of diabetes, education level, and physical activity. Only statistically significant risk factors were retained in the final model.

A simple point system for estimating diabetes risk was derived using the methods described by Sullivan and colleagues [20]. First, continuous variables were organized into categories and reference values for each variable were separately defined. Second, we determined the referent risk-factor profile by assigning the median value in each category and computed how far each category was from the referent in regression units. Third, beta regression coefficients for continuous and categorical variables were obtained and a constant that reflected the increase in risk associated with a 5-year increase in age was set. Fourth, the point score for each category of predictors was estimated using the product of the corresponding regression coefficients and how far the median of each category was from the associated reference group. The point range was calculated based on the points for each predictor.

Once the simple point system was generated, we evaluated its diagnostic capacity on the remnant sample members (this constituted one-third of the overall sample population and was referred to as the “testing sample”). The receiver operating characteristic (ROC) curve was obtained by plotting sensitivity against 1-specificity at each cutoff value. Areas under the receiver operating characteristic curve (AUC) were also calculated for the present risk score and several previously reported risk scores that were developed in eastern Asia [16, 21–23] and in the West [8, 9, 12, 13, 24]. Diagnostic accuracy was assessed by the area under the curve (AUC). C-statistics were used to compare the AUCs. The calibration feature of the prediction scores was estimated using the Hosmer-Lemeshow test, in which a non-significant P value indicates good agreement between observed outcomes and model-based predictions. The optimal cut-off point for each risk score was a value that maximized the sum of sensitivity and specificity. Sensitivity, specificity, positive and negative predictive values, positive and negative likely ratios, and Youden index were also calculated. A two-sided P value <0.05 was considered statistically significant.

## Results

### Baseline characteristics

The current analysis was restricted to 4,132 subjects who presented with complete diabetes data. The baseline characteristics of the 4,132 participants are shown in Table 1. At baseline, 56.6% of the participants were women, 64.9% were illiterate or were educated to primary school level, 75.7% were engaged in regular physical activity, and 47.2% were hypertensive. Compared with men, women had higher BMI, SBP, and hypertension, but lower waist circumference, WHR, education level, and physical activity. Men exhibited significantly higher smoking rates (59.9% versus 0.3%) and consumed more alcohol (40.8% versus 0.8%) than women.

**Table 1:** Baseline characteristics of men and women participated included in the present study (n = 4132).

	Total (n = 4132)	Men (n = 1793)	Women (n = 2339)	*P* value
Age, years	51±11	52±11	51±11	0.011
BMI, kg/m^2^	24.6±3.6	24.3±3.6	24.9±3.6	<0.001
WC, cm	87.1±9.2	88.0±8.8	86.4±9.5	<0.001
WHR	0.90±0.05	0.92±0.05	0.88±0.05	<0.001
SBP, mmHg	138.3±21.4	137.4±20.3	138.9±22.2	0.023
DBP, mmHg	77.7±11.9	77.7±12.1	77.6±11.8	0.755
FPG, mmol/L	5.48 (5.15–5.84)	5.49 (5.14–5.85)	5.46 (5.16–5.83)	0.622
Current smokers, n (%)	1080 (26.1)	1074 (59.9)	6 (0.3)	<0.001
Current drinker, n (%)	749 (18.1)	731 (40.8)	18 (0.8)	<0.001
Education, n (%)				<0.001
Illiterate	567 (13.7)	143 (8.0)	424 (18.1)	
Primary School	2117 (51.2)	754 (42.1)	1363 (58.3)	
Junior high	1321 (32.0)	804 (44.8)	517 (22.1)	
Senior high	127 (3.1)	92 (5.1)	35 (1.5)	
Physical activity, n (%)				<0.001
Low	817 (19.8)	282 (15.7)	535 (22.9)	
Moderate	186 (4.5)	84 (4.7)	102 (4.4)	
High	3129 (75.7)	1427 (79.6)	1702 (72.8)	
Hypertension, n (%)	1950 (47.2)	783 (43.7)	1167 (49.9)	<0.001
Family history of diabetes, n (%)	196 (4.7)	75 (4.2)	121 (5.2)	0.138
Follow-up Diabetes, n (%)	218 (5.3)	82 (4.6)	136 (5.8)	0.077

Data are presented as mean±SD, median (interquartile range) or percent. Chi-square test for categorical variables, the unpaired t test or Mann-Whitney U test for continuous variables. BMI, body mass index; WC, waist circumference; WHR, waist/hip rate; SBP, systolic blood pressure; DBP, diastolic blood pressure; FPG, fasting plasma glucose

### Diabetes incident rates

Of the 4,132 participants who presented without diabetes at the baseline examination, 218 developed diabetes in the period leading up to the 6-year follow-up. Of these cases, 76 (34.9%) were identified by reporting a clinical diagnosis or following the use of anti-diabetic medications and 142 (65.1%) were identified following FPG or HbA1c testing.

### Non-invasive risk model predicting incident diabetes

In the forward stepwise multivariable logistic regression analysis, increased age, BMI, waist circumference, and positive family history of diabetes were significantly associated with incident diabetes (Table 2); however, gender, smoking, alcohol use, physical activity, and education level were not significant risk factors for predicting incident diabetes. Therefore, age, BMI, waist circumference, and positive family history of diabetes were used in the final model. The AUCs were 0.715 (95% CI, 0.672–0.757) and 0.704 (95% CI, 0.669–0.739) in the training and testing samples, respectively.

**Table 2:** Stepwise logistic regression analyses for non-invasive risk factors for incident type 2 diabetes in the present study.

Variables	β-Coefficient	Odd ratios (95%CI)	*P* value
Intercept	-10.6393		<0.001
Age	0.0307	1.03 (1.01–1.05)	<0.001
BMI	0.0564	1.06 (1.01–1.11)	0.013
WC	0.0511	1.05 (1.03–1.08)	<0.001
Family history of diabetes	1.3146	3.72 (2.15–6.46)	<0.001

BMI, body mass index; WC, waist circumference

### Development of risk scores for predicting incident diabetes

As shown in Table 3, a simple point system was developed based on the logistic regression coefficients and reference values for each significant risk factor. The simple non-invasive risk score included age (8 points), BMI (3 points), waist circumference (7 points), and family history of diabetes (9 points). The score ranged from 0 to 27. The AUC of the non-invasive risk score was 0.686 in the testing sample. The scores were manually counted to estimate the risk of diabetes development in individuals analyzed as part of this study. In the present study, 63.0% had a risk of ≤5.0%, 26.5% had a risk of >5.0% and ≤10.0%, and 10.5% had a risk of >10% using this score system.

**Table 3:** Algorithm to estimate risk for incident type 2 diabetes using total points for the non-invasive model with logistic regression analysis in the 2754 participants of the training population.

Risk factor	Reference value (W_ij_)	β_i_	*P* value	β_i_(W_ij_-W_iREF_)	Point_ij_ = β_i_(W_ij_-W_iREF_)/B*
Age, years		0.0307	<0.001		
30–39	34.5(W_1REF_)			0	0
40–49	44.5			0.307	2
50–59	54.5			0.614	4
60–69	64.5			0.921	6
≥70	73.0			1.182	8
Body mass index, kg/m^2^		0.0565	0.013		
<24	22.0(W_2REF_)			0	0
24–27.9	26.0			0.226	1
≥28	30.0			0.405	3
Waist circumference, male/female, cm		0.0511	<0.001		
<80/75	77.0/72.0(W_3REF_)			0	0
80–84.9/75-79.9	82.5/77.5			0.281	2
85–89.9/80-84.9	87.5/82.5			0.537	3
90–94.9/85-89.9	92.5/87.5			0.792	5
≥95/90	99.0/95.0			1.124	7
Family history of diabetes		1.3146	<0.001		
NO	0(W_4REF_)			0	0
YES	1			1.315	9

B = 5*0.0307 = 0.1535

### Comparisons with other prediction risk scores developed in Asian and Western countries

**Fig 2:**
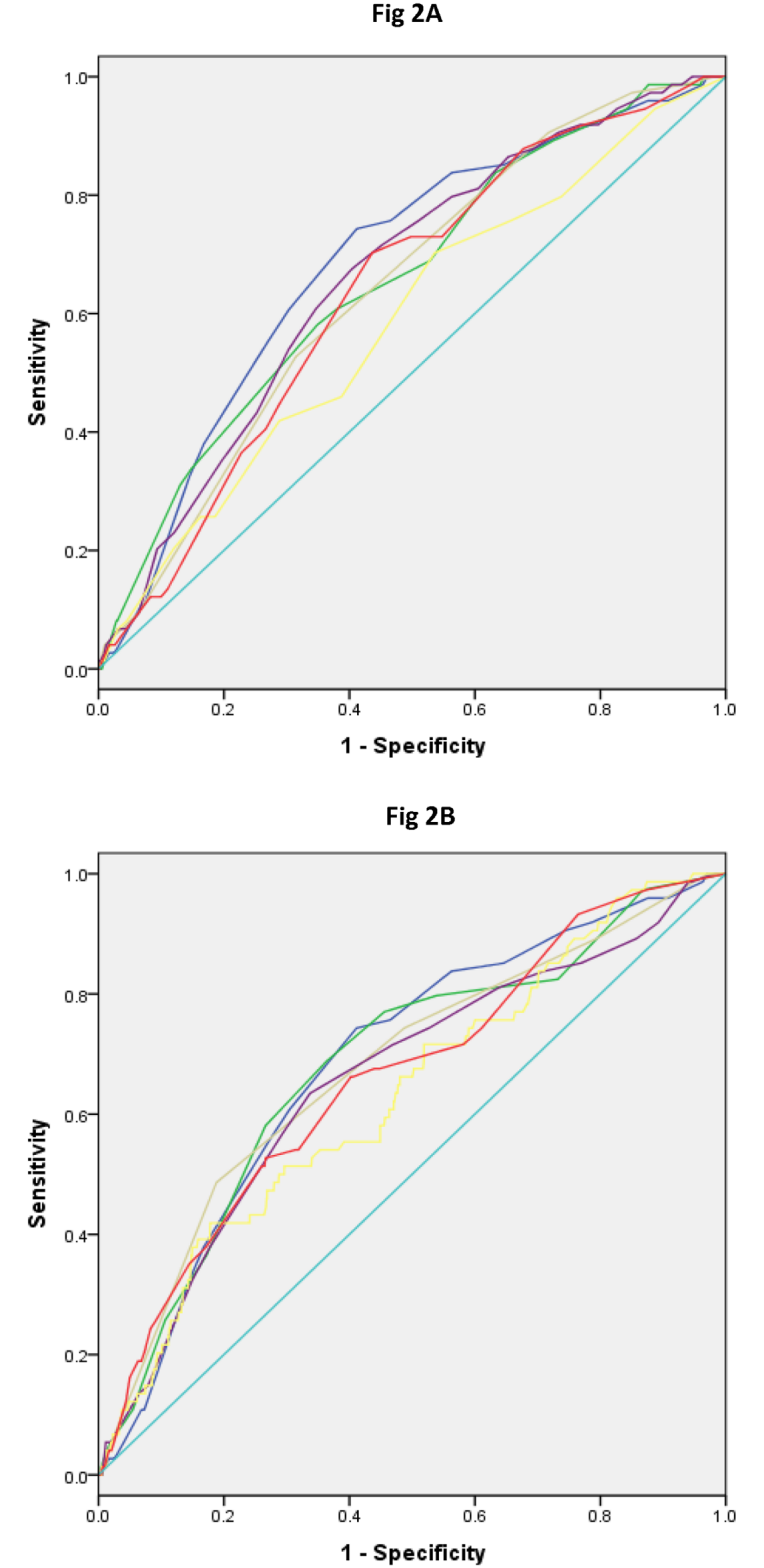
(A) Receiver operating characteristic curves for various scores applied to the validation population in HES in 2006–2013. Blue, current diabetes risk score (AUC, 0.686); Purple, China diabetes risk score (AUC, 0.662); Green, Thai risk score (AUC, 0.656); Grey, Korean risk score (AUC, 0.643); Yellow, Japanese risk score (AUC, 0.584); Red, Qingdao risk score (AUC, 0.636). (B) Receiver operating characteristic curves for various scores applied to the validation population in HES in 2006–2013. Blue, current diabetes risk score (AUC, 0.686); Green, FINDRISC score (AUC, 0.681); Grey, French DESIR score (AUC, 0.677); Purple, AUSDRISK score (AUC, 0.655); Yellow, Cambridge risk score (AUC, 0.632); Red, Framingham risk score (AUC, 0.661).

The present non-invasive Chinese Diabetes Risk Score was compared with ten scores derived from other populations that were applicable to the testing sample (Table 4, Fig 2A & 2B). The present performance of the Non-invasive Chinese Diabetes Score is superior to that of other existing risk scores in terms of AUCs. With an optimal cut-off value of 9, the present non-invasive risk score had the highest Youden index (0.3314), highest sensitivity (74.32%), and highest negative predictive value among all the risk scores.

**Table 4:** Performance of the present Chinese Diabetes Risk Score and other published scores in Asian and Westerner for predicting incident diabetes in validation population.

Scores	Risk factors in the score	AUC	Optimal cutoff value	Sensitivity	Specificity	+LR	-LR	+PV	-PV	Youden index	Hosmer and Lemeshow Test
FINDRISC score (8)	Age, BMI, WC, use of blood pressure medication, history of high blood glucose	0.681(0.656–0.706)	>5	68.92(57.1–79.2)	63.50(60.8–66.1)	1.89(1.6–2.2)	0.49(0.3–0.7)	9.7(7.3–12.5)	97.3(96.0–98.3)	0.3242	0.021
Framingham risk score (9)	age, sex, BMI, parental history of diabetes	0.661(0.635–0.686)	>5.6	52.70(40.7–64.4)	73.37(70.9–75.8)	1.98(1.6–2.5)	0.64(0.5–0.8)	10.1(7.3–13.6)	96.5(95.1–97.5)	0.2607	0.170
AUSDRISK score (12)	age, sex, ethnicity, WC, family history of diabetes, history of high blood glucose, antihypertensive medication, smoking, physical inactivity	0.655(0.629–0.680)	>11	63.51(51.5–74.4)	66.26(63.6–68.8)	1.88(1.6–2.3)	0.55(0.4–0.7)	9.7(7.2–12.6)	97.0(95.6–98.0)	0.2977	0.101
French DESIR score (13)	WC, hypertension, smoking(men), family history of diabetes(women)	0.677(0.652–0.702)	>3	48.65(36.9–60.6)	81.21(79.0–83.3)	2.59(2.0–3.4)	0.63(0.5–0.8)	12.8(9.1–17.3)	96.5(95.3–97.5)	0.2986	0.655
Cambridge risk score (24)	Age, gender, BMI, steroid and antihypertensive medication, smoking, family history of diabetes	0.632(0.606–0.658)	>-1.347	41.89(30.5–53.9)	82.19(80.0–84.2)	2.35(1.8–3.2)	0.71(0.6–0.9)	11.8(8.2–16.3)	96.1(94.8–97.2)	0.2409	0.222
Thai risk score (21)	Age, sex, WC, BMI, hypertension, family history of diabetes	0.656(0.630–0.681)	>8	58.11(46.1–69.5)	65.11(62.5–67.7)	1.67(1.4–2.0)	0.64(0.5–0.8)	8.6(6.3–11.5)	96.5(95.0–97.6)	0.2322	0.260
Korean risk score (22)	Age,smoking, alcohol use, WC, hypertension, family history of diabetes	0.643(0.617–0.668)	>7	52.7(40.7–64.4)	68.56(66.0–71.1)	1.68(1.3–2.1)	0.69(0.5–0.9)	8.7(6.2–11.7)	96.2(94.8–97.4)	0.2126	0.661
Japanese risk score (23)	age, sex, family history of diabetes, smoking and BMI	0.584(0.557–0.610)	>6	70.27(58.5–80.3)	46.40(43.7–49.1)	1.31(1.1–1.5)	0.64(0.4–0.9)	6.9(5.2–9.0)	96.5(93.9–97.7)	0.1667	0.457
Qingdao Diabetes Score (15)	Age, WC, family history of diabetes	0.636(0.610–0.661)	>15	70.27(58.5–80.3)	56.37(53.6–59.1)	1.61(1.4–1.9)	0.53(0.4–0.8)	8.4(6.3–10.8)	97.1(95.6–98.2)	0.2664	0.473
China diabetes risk score (16)	Age, sex, WC, BMI, SBP, family history of diabetes	0.662(0.637–0.687)	>30	67.57(55.7–78.0)	59.59(56.9–62.3)	1.58(1.4–1.8)	0.52(0.4–0.7)	8.2(6.2–10.6)	97.1(95.7–98.2)	0.2715	0.248
the present Chinese Diabetes Risk Score	Age, WC, BMI, family history of diabetes	0.686(0.661–0.710)	>9	74.32(62.8–83.8)	58.82(56.1–61.5)	1.80(1.6–2.1)	0.44(0.3–0.6)	9.3(7.1–11.9)	97.6(96.3–98.5)	0.3314	0.807

BMI, body mass index; WC, waist circumference; SBP, systolic blood pressure

## Discussion

### Main findings

Using information pertaining to age, obesity, and familial diabetes history, we developed a simple non-invasive risk score system that is simple to use and facilitates diabetes predictions in a rural Chinese population containing individuals aged 30 years and over. These values are easily obtained from families and health care clinics in rural areas in China. The new non-invasive Chinese Diabetes Risk Score proved to be more optimal at predicting diabetes than most of the existing risk scores developed in Western and East Asian countries.

### Comparison with other non-invasive diabetes risk scores

To date, most non-invasive risk scores for predicting incident diabetes have been developed in western populations [8–13]. The common risk factors included in these diabetes risk scores were age, sex, obesity, family history of diabetes, hypertension, and lifestyle. The AUCs of these risk scores ranged from 0.724 to 0.857 in the original populations. Recently, Kengne *et al*. [14] externally validated these existing non-invasive risk models and assessed predictive performance variability in European populations. The authors found that these models could be used to identify individuals in the general population at high risk of developing diabetes (C-statistics ranged from 0.76 to 0.81). However, the performance of each model varied with country, age, sex, and adiposity. Moreover, several studies conducted in the Asian population have demonstrated that diabetes risk models developed in western populations performed poorly when used for the detection of undiagnosed diabetes in Asians [22, 25], including Chinese [16]. In the present study, we found that the AUCs of the diabetes risk models developed in western populations were lower in this rural Chinese population than in their original populations. The predominant reason for the lack of transferability associated with these risk scores may be underpinned by the fact that each ethnic group has different and distinctive genetic and environmental characteristics, such as body shape, diets, culture, and other lifestyle factors. Therefore, an ethnic- or country-specific diabetes risk score may be required to predict the potential for diabetes development.

Recently, several non-invasive risk scores for detecting undiagnosed diabetes or incident diabetes have been developed for Asian [21–23] and Chinese populations [15, 16]. The Asian diabetes risk scores utilized common risk factors (including age, obesity, and family history of diabetes) and generated more optimal results for the original population samples compared to the current rural Chinese population. Compared with the Chinese Diabetes Risk Score derived from data generated from the China National Diabetes and Metabolic Disorders Study [16] (S1 Reference), our diabetes risk score does not include systolic blood pressure. Additionally, the newly generated diabetes risk score performed more optimally in the rural Chinese population that was analyzed as part of this study. Therefore, the latter risk score may be easier to use since a blood pressure monitor is not available to most families in rural areas. Compared with the Qingdao diabetes risk score derived from Chinese living urban community in a cross-sectional study [15] (S1 Reference), which was developed for screening undiagnosed diabetes, the present Chinese diabetes risk score was developed for predicting incident diabetes in a village-based cohort study. Further, the performance of the Qingdao Diabetes Score is inferior to that of most existing diabetes risk scores in terms of AUCs when used for predicting incident diabetes in current rural Chinese population (The AUC was 0.636 in the testing sample). According to a recent national survey in China [2], it is estimated that the prevalence of pre-diabetes was about 50% in 2010. Therefore, a simple non-invasive risk score for predicting incident diabetes (as described here) could be introduced to rural families and primary health care providers. This would help to identify individuals with an increased risk of developing diabetes in rural China.

### Strengths and limitations

To our knowledge, this is the first cohort study carried out with the aim of developing a non-invasive risk score for predicting incident diabetes in a rural Chinese population. However, there are some limitations associated with this study. First, a total of 6,830 participants took part in the HES in 2006–2007, and the follow-up survey was conducted in 2012–2013. We excluded 2,698 individuals for numerous reasons, thereby leaving 4,132 participants for the present analysis. Therefore, it may be theorized that selection bias influenced the results generated during this study, and this is a fairly small study compared to previous much larger studies [10–12, 16, 22–23]. Second, external validation was not performed because of a lack of data from other similarly designed studies in rural China [26]. Third, participants with FPG levels ≥5.6 mmol/l and <7.0 mmol/l did not receive an oral glucose tolerance test to confirm the presence of diabetes. This is likely to have led to some error in estimating the risk of diabetes, thereby affecting the performance of our model. However, according to a recent national survey conducted in China, approximately 85% of newly diagnosed diabetes patients could be identified, by combining FPG and HbA1c values [2]. Lastly, the discriminatory capacity of our simple risk score was moderate (the AUC was 0.686), and somewhat lower than that of other risk scores from other populations.

### Conclusions

In this population-based cohort study in rural China, a simple non-invasive risk score for predicting incident diabetes was developed based upon age, BMI, WC, and family history of diabetes. This score system could be introduced to rural families and primary health care providers, and would help to identify individuals with a high risk of developing diabetes. It is hoped that this system could improve disease prevention and provide the information required to implement treatment strategies for rural Chinese populations.

## Supporting information

S1 Dataset (SAV)

S1 Reference Nonlaboratory-based risk assessment algorithm for undiagnosed type 2 diabetes developed on a nation-wide diabetes survey. (PDF)

S2 Reference A simple Chinese risk score for undiagnosed diabetes. (PDF)
