# Supplementary material for: A non-invasive risk score for predicting incident diabetes among rural Chinese people: A village-based cohort study
Source: PLoS One. 2017 Nov 2;12(11):e0186172. doi: 10.1371/journal.pone.0186172 (PMC5667808; doi:10.1371/journal.pone.0186172)
Supplement: S2 Reference — (PDF) [file pone.0186172.s003.pdf]

DOI: 10.1111/j.1464-5491.2010.02943.x

# Original Article: Epidemiology

# A simple Chinese risk score for undiagnosed diabetes

W. G. Gao*†‡, Y. H. Dong‡, Z. C. Pang§, H. R. Nan¶, S. J. Wang§, J. Ren§, L. Zhang*†‡, J. Tuomilehto*† and Q. Qiao*†

*Department of Public Health, University of Helsinki, †Diabetes Unit, Department of Chronic Disease Prevention, National Institute for Health and Welfare, Helsinki, Finland, ‡Qingdao Endocrinology and Diabetes Hospital, §Department of Non-communicable Disease Prevention, Qingdao Municipal Centre for Disease Control and Prevention, Qingdao and ¶Hong Kong Institute of Diabetes and Obesity, Hong Kong, China

*Accepted 21 December 2009*

## Abstract

**Aims** A diabetes risk score for screening undiagnosed diabetes was constructed and validated in Chinese adults.

**Methods** Two consecutive population-based diabetes surveys among Chinese adults aged 20–74 years were conducted in 2002 ($n$ = 1986) and 2006 ($n$ = 4336). Demographic and anthropometric measures were collected following similar procedures. Standard 2-h 75-g oral glucose tolerance tests (OGTTs) were performed to diagnose diabetes in both surveys. Fasting capillary plasma glucose (FCG) and glycated haemoglobin ($HbA_{1c}$) were also measured together with the OGTTs on the same day of the 2006 survey. Beta coefficients estimated using logistic regression analysis derived from data of the 2002 survey were used to develop the risk assessment algorithm. The performance of the algorithm was validated in the study population of the 2006 survey.

**Results** Of all the variables tested, waist circumference, age and family history of diabetes were significant predictors of diabetes and were used to construct the risk assessment score. The score, ranging from 3 to 32, performed well when applied to the study population of the 2006 survey. The area under the receiver operating characteristic curve was 67.3% (95% CI, 64.9–69.7%) for the score, while it was 76.3% (73.5–79.0%) for FCG alone and 67.8% (64.9–70.8%) for $HbA_{1c}$ alone. At a cut-off point of 14, the sensitivity and specificity of the risk score were 84.2% (81.0–87.5%) and 39.8% (38.2–41.3%).

**Conclusions** The risk score based on age, waist circumference and family history of diabetes is efficient as a layperson-oriented diabetes screening tool for health promotion and for population-based screening programmes.

Diabet. Med. 27, 274–281 (2010)

**Keywords** risk assessment, score, undiagnosed diabetes

**Abbreviations** AUC, area under the receiver operating characteristic curve; BMI, body mass index; FCG, fasting capillary plasma glucose; $HbA_{1c}$, glycated haemoglobin; NRI, net reclassification improvement; OGTT, oral glucose tolerance test; ROC, receiver operating characteristic; SBP, systolic blood pressure

## Introduction

Since 1999, 14 screening scores (or questionnaires) for screening Type 2 diabetes based on demographic, anthropometric and clinical information have been established and validated in different populations and suggested to be a useful first-line screening test [1–14]. Some of these scores also include lifestyle issues such as dietary factors and physical activity levels [4–7,10].

*Correspondence to*: Qing Qiao, Department of Public Health, University of Helsinki, Helsinki, Finland. Mannerheimintie 172, KTTL, 6krs, PL 41, FIN-00014 Helsinki, Finland. E-mail: qing.qiao@thl.fi

Unlike blood glucose or glycated haemoglobin ($HbA_{1c}$), the self-administrated risk score (questionnaire) does not require blood sampling and laboratory measurements. It is also cheap and can be used in a population-based screening programme for health promotion and prevention, particularly in the less-developed areas.

Currently, most of the self-administrated risk scores (questionnaires) were developed in Caucasian populations, with only a few in Asians. In this study, we report a simple self-administrated diabetes risk score and its performance for screening for undiagnosed diabetes in Chinese adults living in Qingdao, China.



© 2010 The Authors.
Journal compilation © 2010 Diabetes UK. 

## Patients and Methods

### Population constructing the risk score

A cross-sectional population-based survey was performed in 2002 in an urban community in Qingdao city, China [15]. A stratified random cluster sampling procedure was employed to recruit the study subjects. The community, named Zhanshan, has eight street blocks. From each street block, 300 or 400 individuals (20–74 years old) were randomly selected and 2600 individuals were invited to take part. Of these, 2156 participated in the survey, giving a response rate of 82.9%.

### Population validating the risk score

A baseline diabetes prevalence survey was conducted in 2006 in Qingdao before the Qingdao Diabetes Prevention Program (Qd-DPP) was started. The prevention programme targets the entire population of two urban (Shinan and Shibei) and two rural districts (Jiaonan and Huangdao). A random population sample was recruited from the four targeted districts and two geographically matched control districts (Sifang and Jimo). Five communities (or villages) from each of the six districts and 200–250 residents (35–74 years old) from each community (or village) were randomly selected. Six thousand, one hundred randomly selected individuals were invited and 5355 individuals took part in the survey, giving a response rate of 87.8%.

Both the surveys were approved by the local Ethics Committee and Qingdao Health Administrative Bureau. Verbal or written consent was obtained from all participants prior to data collection.

### Methods

The survey procedure, anthropometric measures and survey questionnaire were similar in both surveys, but the 2006 questionnaire contained more questions. "The surveys were carried out in local health stations or community clinics near the participants' residential areas. Participants were interviewed and the study questionnaires completed on site by trained doctors or nurses". Information about diabetes in parents, siblings and/or offspring was collected with comparable questionnaires. Leisure time physical activity during the last 12 months was recorded as: (i) sedentary (no leisure time physical activity), (ii) light (relaxing walking outside, taiji, etc.), (iii) moderate (jogging, brisk walking, bowling, social dancing, etc.) and (iv) strenuous (running, gymnastics, swimming, badminton, tennis, table tennis, etc.). In the 2006 survey, information on frequency and duration of the exercise was also recorded. Participants were divided into non-smokers, including never smokers and ex-smokers, and current smokers if individuals smoked daily for at least 6 months regardless of the amount and type of smoking.

Height and weight were measured with participants wearing light clothes and without shoes. Body mass index (BMI) was then calculated by dividing weight in kilograms (kg) by height in metres squared ($m^2$). Waist circumference was measured at the mid-point between the rib cage and iliac crest, and the hip circumference at the maximal horizontal girth between the waist and thigh. Three consecutive blood pressure readings, at least 30 s apart, were taken from the right arm of seated participants, and the average of the three readings was used in subsequent data analysis.

All the participants were invited to have a standard 75-g oral glucose tolerance test (OGTT), except those with a prior history of diabetes. Plasma glucose concentration was determined by the glucose oxidase method in the same central clinical laboratory of Hiser Medical Centre (Qingdao, China). Fasting capillary plasma glucose (FCG) test (Bayer Brio Meter Kit; Bayer HealthCare LLC, Mishawaka, IN, USA) and $HbA_{1c}$ (Olympus AU640 Automatic Analyser, Olympus Corporation, Tokyo, Japan; Tina-quant Hemoglobin $A_{1c}$, BM/Hitachi Reagent Kit, Roche, Roche Diagnostic (Shanghai) LIM, Shanghai, China) were also measured on the same day as the OGTTs in the 2006 survey.

One hundred and seventy participants from the 2004 survey and 805 from the 2006 survey were excluded from the current data analysis because of missing data. In addition, 331 individuals with a prior history of diabetes were also excluded from the 2006 survey in order to assess the ability of the derived risk score to identify undiagnosed diabetes.

Thus, 1986 participants in the 2002 survey and 4336 in the 2006 survey were included in the current data analysis.

### Classification of individuals

Diabetes was defined according to the 2006 World Health Organization (WHO)/International Diabetes Federation (IDF) criteria [16]. Participants reporting a prior history of diabetes were considered as having known diabetes. In the individuals without known diabetes, undiagnosed diabetes was determined if he/she had a fasting plasma glucose ≥ 7.0 mmol/l and/or a post-challenged plasma glucose ≥ 11.1 mmol/l. The term 'pre-diabetes' was used for those without diabetes but with a fasting plasma glucose of 6.1–6.9 mmol/l and/or a post-challenged plasma glucose of 7.8–11.0 mmol/l.

### Statistical analysis

The risk score was derived from the data of the 2002 survey. Candidate risk factors including age, family history of diabetes, smoking status, physical activity, BMI, waist circumference and systolic blood pressure were fitted into a logistic regression model one by one. Variables which were statistically significant ($P < 0.05$) in the univariate analysis were further fitted into the final multivariate logistic regression model using the backward stepwise LR method. The Hosmer–Lemeshow test was used to examine how well the predicted prevalence matched the observed prevalence. A large $P$-value indicates a good match. The net reclassification improvement (NRI) was also calculated

© 2010 The Authors.

according to the method of Pencina *et al.* [17]. Using the estimated probability of having diabetes, individuals were classified into categories of ≤ 10.9%, 11.0–15.2%, 15.3–19.7% and ≥ 19.8% in men and of ≤ 7.5%, 7.6–11.9%, 12.0–17.7% and ≥ 17.8% in women. These categories were used in NRI analysis. The log–likelihood ratio test was performed to check whether adding a variable to a model improved the model prediction. Beta coefficients derived from the final multivariate logistic regression model were used to calculate the diabetes risk score.

The validation of the risk score derived from the data of the 2002 survey was examined in the population of the 2006 survey. A receiver operating characteristic (ROC) curve was plotted for the score against the presence of undiagnosed diabetes. The optimal cut-off point was identified as the coordinate closest to the y intercept (0, 1) of the ROC curve, and it is at this point that the sum of the sensitivity and the specificity is maximal. C-statistics were used to compare the area under the ROC curve (AUC). All the statistical analyses were performed using SPSS for Windows 15.0 (SPSS Inc., Chicago, IL, USA), except for C-statistic, which was performed using Stata 8.2 (Stata Corp., College Station, TX, USA).

## Results

The characteristics of the participants are shown in Table 1. Compared with the participants in the 2002 survey, those in the 2006 survey were younger, but physically less active, had higher blood pressure and glucose levels and were more likely to smoke. Because of the inclusion of the rural participants in the latter survey, both the BMI and waist circumference were lower in the 2006 survey than those in 2002.

### Development of the risk score

Logistic regression analysis was performed to estimate the strength of the association of the candidate factors to the presence of undiagnosed diabetes. Age, waist circumference and diabetes in parents and/or siblings were independently associated with the presence of undiagnosed diabetes in both men and women (Table 2). Waist circumference appeared to be a strong modifiable risk factor for Type 2 diabetes in this study population as shown by the log–likelihood ratio test (Table 2). Systolic blood pressure (SBP) was associated with the presence of undiagnosed diabetes in females only (Table 2); however SBP did not improve the model reclassification [net gain in reclassification proportion of 0.023 in men ($P$ = 0.75) and 0.015 in women ($P$ = 0.89) calculated using NRI] when it was added to the model including age, family history of diabetes and waist circumference. SBP was thus not included in the final model, in order to make the final model similar in women and men and easy to use by a layperson (Table 2). The Hosmer–Lemeshow test showed that the predicted risk of the final model compared well with the observed risk ($\chi^2$ = 6.23, $P$ = 0.62 in men; and $\chi^2$ = 10.36, $P$ = 0.24 in women). Based on the beta coefficient of the final model (Table 2), the risk score was constructed as shown in Table 3. The optimal

**Table 1** Characteristics of participants without a prior history of diabetes in the two surveys

| | 2002 survey | | 2006 survey | |
|---|---|---|---|---|
| | Men | Women | Men | Women |
| No. of participants | 741 | 1245 | 1687 | 2649 |
| Age (years) | 54 ± 11.9 | 52 ± 11.8 | 50 ± 10.9 | 49 ± 10.1 |
| Body mass index (kg/m$^2$) | 26.5 ± 3.5 | 26.1 ± 3.9 | 25.7 ± 3.2 | 25.8 ± 3.6 |
| Waist circumference (cm) | 90.2 ± 9.4 | 83.5 ± 10.0 | 87.6 ± 9.7 | 82.0 ± 9.7 |
| Hip circumference (cm) | 99.5 ± 6.8 | 98.9 ± 7.2 | 98.8 ± 7.2 | 99.0 ± 7.5 |
| Systolic blood pressure (mmHg) | 130 ± 20 | 129 ± 22 | 135 ± 20 | 132 ± 23 |
| Diastolic blood pressure (mmHg) | 85 ± 11 | 82 ± 11 | 87 ± 12 | 84 ± 12 |
| Fasting plasma glucose (mmol/l) | 5.5 ± 1.26 | 5.5 ± 1.26 | 5.6 ± 1.25 | 5.5 ± 1.21 |
| Two-h post-load glucose (mmol/l) | 6.0 ± 1.59 | 6.1 ± 1.46 | 6.6 ± 1.44 | 6.9 ± 1.39 |
| Fasting capillary glucose (mmol/l)* | — | — | 5.9 ± 1.22 | 5.9 ± 1.24 |
| $HbA_{1c}$ (%) | — | — | 5.6 ± 1.14 | 5.5 ± 1.13 |
| Triglyceride (mmol/l) | 1.5 ± 1.73 | 1.3 ± 1.67 | 1.2 ± 1.77 | 1.1 ± 1.63 |
| High-density lipoprotein cholesterol (mmol/l) | 1.5 ± 1.20 | 1.5 ± 1.21 | 1.6 ± 1.30 | 1.6 ± 1.27 |
| Diabetes in parent or sibling (%) | 19.6 | 19.8 | 15.3 | 18.5 |
| Current smoker (%) | 37.4 | 1.0 | 54.2 | 2.2 |
| Physical activity (%) | | | | |
| Sedentary | 24.7 | 17.3 | 65.9 | 64.3 |
| Light | 49.3 | 59.2 | 22.6 | 23.9 |
| Moderate or heavy | 21.9 | 21.0 | 8.4 | 7.1 |
| Undiagnosed diabetes (%) | 10.9 | 9.1 | 12.3 | 10.3 |

Data are mean ± SD or percentage.
*$n$ = 1555 in men and 2393 in women.
$HbA_{1c}$, glcyated haemoglobin; SD, standard deviation.

© 2010 The Authors.

**Table 2** The risk factors and the beta coefficient derived from the logistic regression analyses based on the data of the 2002 survey

| | Univariate model | | Final model | | |
|---|---|---|---|---|---|
| | β coefficient (SE) | Odds ratio (95% CI) | β coefficient (SE) | Odds ratio (95% CI) | $\chi^2$ (*P* value for 1 d.f.)† |
| Men | | | | | |
| Age (year) | 0.03 (0.01) | 1.03 (1.01–1.05) | 0.03 (0.01) | 1.03 (1.01–1.05) | 10.75 (0.001) |
| BMI (kg/m$^2$) | 0.06 (0.03) | 1.06 (1.01–1.12) | — | | |
| Waist (Chinese chi)* | 0.12 (0.03) | 1.12 (1.05–1.20) | 1.09 (0.33) | 2.98 (1.56–5.68) | 11.44 (0.001) |
| Systolic blood pressure, (mmHg) | 0.01 (0.00) | 1.01 (1.00–1.02) | — | — | |
| Diabetes in parents or siblings | 0.60 (0.23) | 1.82 (1.16–2.84) | 0.73 (0.24) | 2.07 (1.30–3.29) | 8.91 (0.003) |
| Leisure time physical activity (moderate) | — | 1 | — | — | |
| Sedentary | 0.16 (0.32) | 1.18 (0.63–2.20) | — | — | |
| Light | 0.50 (0.27) | 1.66 (0.97–2.83) | — | — | |
| Current smoker (vs. non-smoker) | –0.09 (0.21) | 0.91 (0.60–1.38) | — | — | |
| Women | | | | | |
| Age (year) | 0.05 (0.01) | 1.05 (1.03–1.06) | 0.04 (0.01) | 1.04 (1.02–1.06) | 22.16 (0.001) |
| BMI (kg/m$^2$) | 0.07 (0.02) | 1.07 (1.03–1.11) | — | — | |
| Waist (Chinese chi)* | 0.14 (0.03) | 1.15 (1.09–1.20) | 0.86 (0.28) | 2.36 (1.35–4.12) | 9.11 (0.001) |
| Systolic blood pressure (mmHg) | 0.02 (0.00) | 1.02 (1.02–1.03) | — | — | |
| Diabetes in parents or siblings | 0.51 (0.19) | 1.67 (1.15–2.42) | 0.82 (0.20) | 2.27 (1.53–3.38) | 15.38 (0.001) |
| Leisure time physical activity (moderate) | — | 1 | — | — | |
| Sedentary | –0.05 (0.28) | 0.95 (0.55–1.65) | — | — | |
| Light | 0.10 (0.21) | 1.11 (0.73–1.69) | — | — | |
| Current smoker (vs. non-smoker) | 0.60 (1.04) | 1.83 (0.24–14.18) | — | — | |

*1 Chinese chi ≈ 33 cm.
†Value of $\chi^2$ calculated with log–likelihood test.
BMI, body mass index; CI, confidence interval; SE, standard error.

cut-off value of the risk score for undiagnosed diabetes was 17 in men and 14 in women. Among all the participants with undiagnosed diabetes in the 2002 survey, 97.5% of men and 86.8% of women had a risk score ≥ 14; 85.0% of men and 69.5% of women had a score ≥ 17, respectively.

### Validation of the risk score

The risk score derived from the data of the 2002 survey was assessed by applying the score to the population of the 2006 survey. The ROC curves for the risk score, FCG and $HbA_{1c}$ are plotted in Fig. 1. The discrimination of the score was compared with FCG and $HbA_{1c}$. The score gave an AUC of 63.5% (95% CI, 59.1–67.9%) in men and 68.9% (63.6–72.4%) in women for detecting undiagnosed diabetes, which was slightly lower than that of FCG ($P < 0.001$) but not different from that of $HbA_{1c}$ ($P > 0.20$). Given the same sensitivity, the specificities of the FCG test were highest; however, there was no significant difference between $HbA_{1c}$ and the risk score (Table 4).

The AUC of the risk score for pre-diabetes was 61.2% (57.9–64.5%) in men and 63.2% (60.7–65.7%) in women. For FCG, the AUC was 62.4% (59.0–65.7%) in men and 63.2% (60.5–65.8%) in women. At a cut-off value of ≥ 14, the sensitivity and specificity of the risk score to identify pre-diabetes was 85.6% (83.9–87.4%) and 21.1% (19.0–23.1%) in men and 75.5% (73.8–77.2%) and 43.6% (41.6–45.6%) in women, respectively.

**Table 3** The risk score sheet based on age, sex, waist circumference and diabetes in parents or siblings

| Waist (Chinese chi*) | | | |
|---|---|---|---|
| Men | Score | Women | Score |
| ≤ 2.3 | 1 | ≤ 2.0 | 1 |
| 2.4–2.6 | 4 | 2.1–2.3 | 3 |
| 2.7–2.9 | 8 | 2.4–2.6 | 6 |
| ≥ 3.0 | 12 | ≥ 2.7 | 9 |
| Age (years) | Score | | |
| ≤ 35 | 1 | | |
| 36–45 | 3 | | |
| 46–55 | 6 | | |
| 56–65 | 9 | | |
| ≥ 65 | 12 | | |
| Diabetes in parents and/or siblings | | | Score |
| Negative | | | 1 |
| Positive | | | 8 |
| Score range | | | 3–32 |

*1 Chinese chi ≈ 33 cm.

© 2010 The Authors.

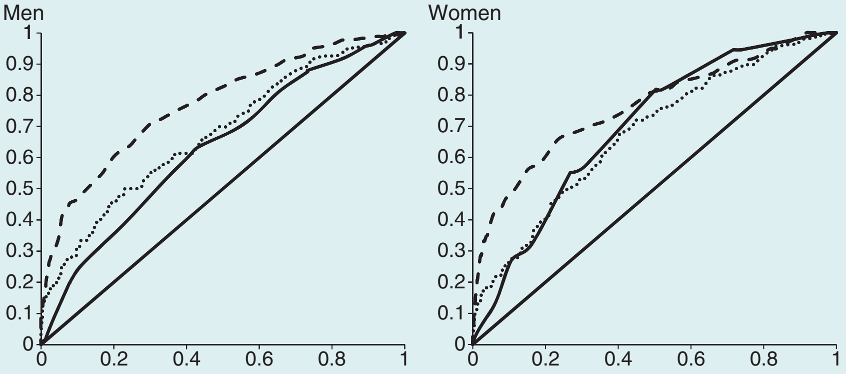


**FIGURE 1** Receiver operating characteristic (ROC) curve of the risk score (——), fasting capillary blood glucose test (FCG, — —) and glycated haemoglobin $A_{1c}$ ($HbA_{1c}$, ·······) in predicting undiagnosed diabetes in men and women. The area under the ROC curve was 63.5% (95% CI, 59.1–67.9%) in men and 68.9% (63.6–72.4%) in women for the risk score, 76.9% (72.9–80.9%) in men and 76.0% (72.2–79.8%) in women for FCG and 68.0% (63.6–72.4%) in men and 67.5% (63.6–71.4%) in women for $HbA_{1c}$.

**Table 4** Sensitivity (%), specificity (%) and positive predictive value (%) of the risk score, as compared with fasting capillary plasma glucose (FCG) test and glycated haemoglobin ($HbA_{1c}$) at different cut-off points, for predicting undiagnosed diabetes

| | Number (%) | Sensitivity (95% CI) | Specificity (95% CI) | Positive predictive value (95% CI) |
|---|---|---|---|---|
| Men | | | | |
| Risk score ≥ 14 | 1266 (75%) | 87.0 (82.2–91.7) | 27.4 (25.0–29.7) | 14.4 (12.4–16.3) |
| Risk score ≥ 17* | 773 (46%) | 64.1 (57.3–70.8) | 56.7 (54.1–59.3) | 17.2 (15.5–18.9) |
| FCG ≥ 6.25 mmol/l* | 645 (35%) | 71.0 (64.3–77.7) | 69.7 (67.2–72.2) | 24.7 (22.4–27.1) |
| FCG ≥ 6.10 mmol/l | 648 (42%) | 74.0 (67.8–80.2) | 62.9 (60.3–65.4) | 21.8 (20.0–23.7) |
| FCG ≥ 5.60 mmol/l | 963 (62%) | 86.5 (81.6–91.3) | 41.5 (38.9–44.1) | 17.2 (16.2–18.2) |
| $HbA_{1c}$ ≥ 5.9%* | 399 (26%) | 50.0 (42.6–57.4) | 76.9 (74.6–79.2) | 23.3 (20.1–26.5) |
| Women | | | | |
| Risk score ≥ 14* | 1462 (55%) | 80.7 (75.7–85.7) | 47.5 (45.4–49.6) | 15.3 (13.4–17.2) |
| Risk score ≥ 17 | 883 (33%) | 55.0 (48.7–61.4) | 69.0 (67.0–70.9) | 16.9 (15.1–18.8) |
| FCG ≥ 6.55 mmol/l* | 1003 (26%) | 65.9 (59.5–72.3) | 76.4 (74.5–78.3) | 24.3 (22.0–26.5) |
| FCG ≥ 6.10 mmol/l | 1009 (42%) | 71.8 (66.1–77.6) | 61.1 (59.1–63.2) | 17.5 (16.1–18.9) |
| FCG ≥ 5.60 mmol/l | 1489 (62%) | 84.5 (79.9–89.1) | 40.2 (38.2–42.3) | 14.0 (13.2–14.7) |
| $HbA_{1c}$ ≥ 5.4%* | 1089 (47%) | 72.0 (66.0–78.0) | 55.3 (53.1–57.5) | 15.6 (14.4–16.8) |

*The optimal cut-off values.
CI, confidence interval.

### Comparison of the current risk score with other existing scores

We also used other previously published scores which were derived from other populations to our 2006 study population. Eight such risk scores [1–5,7,8,11] were applicable to our data and, thus, validated. The performance of these scores was poorer in our population of the 2006 survey than in their original study populations as shown by the lower AUCs (Table 5). None of the existing risk scores had a significantly larger AUC than the simple risk score developed in this study.

## Discussion

In the current study, the simple risk score constructed with age, sex, waist circumference and history of diabetes in parents and/or siblings showed an adequate performance for undiagnosed diabetes. Although its performance was inferior to that of FCG, it was similar to that of $HbA_{1c}$. With regard to the classification of the pre-diabetes, the current diabetes risk score was as accurate as the FCG test.

The proportion of undiagnosed diabetes is high in China, accounting for 50–80% of the diabetic population [15,18–20]. Considering the large number of individuals with diabetes, the low diabetes awareness and the high medical cost of diabetes care in China, it is politically and practically important to find a simple risk assessment tool to identify undiagnosed diabetes and pre-diabetes in order to provide individuals with early treatment or lifestyle intervention to reduce the burden of the disease. Currently, there is no routine clinical record available to identify high-risk individuals in China. The FCG test had a


© 2010 The Authors.

**Table 5** Risk assessment scores (questionnaires) developed in Caucasians or other Asian populations and their performance in the Chinese population of the 2006 Qingdao survey

| Risk assessment scores and reference | Risk factors involved | Optimal cut–off value (range) | Area under the ROC curve (95% CI), (%) | | | Sensitivity (95% CI) at the optimal cut-off value, (%) | | Specificity (95% CI) at the optimal cut-off value, (%) | |
|---|---|---|---|---|---|---|---|---|---|
| | | | In original population | In Chinese in Qingdao | *P* value* | In original population | In Chinese in Qingdao | In original population | In Chinese in Qingdao |
| Cambridge risk model [3] | Age, sex, drug-treated hypertension, corticosteroid treatment, family history of diabetes, BMI and smoking | 0.199 | 80.0 (68–91) | 67.6 (65.2–69.3) | 0.91 | 77.3 (54.6–92.2) | 42.2 (37.8–46.6) | 72.0 (68.0–76.0) | 79.5 (78.3–80.8) |
| Danish risk score [5] | Age, sex, BMI, known history of hypertension, diabetes in parents, physical activity at leisure time | 29 (0–60) | 80.4 (76.5–83.8) | 69.0 (66.6–71.3) | 0.10 | 79.3 (71.4–86.3) | 55.1 (50.6–59.5) | 68.7 (67.1–70.3) | 72.1 (70.7–73.6) |
| Indian risk score [7] | Age, sex, family history of diabetes, BMI, waist circumference, physical activity | 21 (0–42) | 73.2 (70.2–76.1) | 67.5 (65.1–70.0) | 0.91 | 76.6 (70.9–81.7) | 96.1 (94.3–97.9) | 59.9 (58.5–61.3) | 18.7 (17.4–19.9) |
| Rotterdam study [1] | Age, sex, drug-treated hypertension, BMI | 6 (0–22) | 68.0 (64–72) | 63.1 (60.5–65.8) | 0.002 | 78.0 | 18.8 (15.3–22.3) | 55.0 | 90.4 (89.5–91.3) |
| Finnish risk score [4] | Age, BMI, waist, drug-treated hypertension, physical inactivity, daily consumption of vegetables, fruit or berries | 9 (0–20) | 85.0 | 66.5 (63.7–69.3) | 0.92 | 78.0 (71.0–84.0) | 39.5 (34.6–44.4) | 77.0 (76.0–79.0) | 80.4 (79.0–81.7) |
| DESIR risk score [11] | Waist circumference, smoking, hypertension, family history of diabetes | Not available (0–5) | 73.3 in men, 83.9 in women | 55.4 in men, 67.9 in women | < 0.001 in men, 0.13 in women | – | – | – | – |
| Thai risk score [8] | Age, sex, BMI, waist circumference, hypertension, family history of diabetes | 6 (0–17) | 74.0 (71–78) | 66.2 (63.7–68.6) | 0.15 | 77.0 | 86.8 (83.7–89.8) | 60.0 | 32.6 (31.1–34.1) |
| ADA recommendation [3]* | Delivery of a baby weighing > 4 kgs, diabetes in parents or siblings, BMI, age, physical activity | 10 (0–27) | 71.0 | 66.1 (63.6–68.6) | 0.18 | 80.0 | 39.3 (34.9–43.7) | 34.6 | 80.1 (78.9–81.4) |
| Qingdao diabetes risk score | Age, waist circumference, diabetes in parents or siblings | 14 (3–32) | 64.2 (60.3–68.0) | 67.3 (64.9–69.7) | – | 88.7 (84.2–93.1) | 84.2 (81.0–87.5) | 27.1 (25.0–29.2) | 39.8 (38.2–41.3) |

*Compared with Qingdao diabetes risk score.
ADA, American Diabetes Association; BMI, body mass index; CI, confidence interval; ROC, receiver operating characteristic.

© 2010 The Authors.

slightly better performance in screening for diabetes than the simple risk score but portable glucose meters are less affordable and are not available to most community dwellers in China. In China, most glucose tests are performed in hospitals at the request of a doctor. In addition, the difficulty of confirming fasting and of performing invasive blood sampling limits its usage as a mass screening tool. It is widely believed by Chinese poeple, particularly the elderly and those from rural populations, that drawing a blood sample will damage the essential functions of the body. Unless absolutely necessary, people do not want to have a glucose test performed. $HbA_{1c}$ is an indicator of long-term glucose levels. The $HbA_{1c}$ test does not require fasting and can be taken at any time of day. It has better reproducibility and is easier to perform than the OGTT. Recently, it has been recommended to use $HbA_{1c}$ for both screening and diagnosis of diabetes [21,22]. However, the current study does not support the use of $HbA_{1c}$ in mass screening in the general population. Considering it is the most expensive test, $HbA_{1c}$ as a mass screening tool may not be suitable in low-income areas with relatively limited resources for medical care, unless the cost decreases to an affordable level in the future.

Different risk assessment tools based on demographic information and clinical measurements had been developed mostly for Caucasians [1–5,7,8,11]. However, these existing risk assessment tools did not perform better in this Chinese population than in their original deriving populations and were not superior to the simple Chinese diabetes risk score. This might be because both genetic and environmental determinants differ between different ethnic groups; for example, body size, diet, lifestyle, climate and response to the same external challenges are all different. This limits the generalization of risk assessment tools from their original country to other countries or areas.

The merits of the current Chinese risk score are: it was developed in the Chinese population; it is simpler than existing risk scores; it is sensitive; and the information required by the risk score is all self-measurable by a layperson. Obesity, indicated by waist circumference, is a strong modifiable risk factor for diabetes and inclusion of waist circumference in the risk score can educate people to reduce weight or prevent further weight increase. Almost all adult Chinese people know their own waist circumference as it is required when purchasing trousers. We have transferred the unit of waist circumference from centimetre to Chinese chi, which will facilitate its use in China. Thus, the current risk score is truly layperson oriented. The omission of SBP from the final model did not result in the loss of prediction but simplified the use of the score in a lay population where blood pressure measurement is not available to most Chinese families. The current risk score has been widely used in the Qingdao Diabetes Prevention Program since 2006. It was publicized through the local media, such as newspapers, Internet, leaflets and radio, and distributed by community clinics to the local residents door to door. The prevention programme was also introduced to local schools and the school students were educated and encouraged to take the risk score back to their homes and neighbours. At the present time, it has reached over 912 000 citizens. Approximately 270 911 people with a risk score $\geq 14$ have returned to the local community clinics, undergone glucose tests and registered in the prevention programme for lifestyle counselling. The other strength of the current study is that the diabetes risk score was constructed and validated in independent population-based studies. In addition, the methodology and procedures of the two studies was similar.

The weakness of the study is that the current risk score might not accurately predict the risk of future development of diabetes because it was based on a cross-sectional study. Thus, it must be assessed in prospective studies in the Chinese population. In spite of its simplicity, some individuals require the help of family members or a community doctor to use the score. The completed information is checked by a community doctor or nurse before a glucose test is prescribed. Considering the low positive predictive value, it is important to correctly interpret a positive result in a timely fashion so that an individual does not worry unnecessarily. It should be borne in mind that the score is only a first-line screening tool, not a diagnostic test. The cost-effectiveness of mass screening using the risk score is not known and awaits analysis of the outcomes of the Qingdao Diabetes Prevention Program.

In conclusion, the diabetes risk score derived in Qingdao China had reasonable performance for screening for undiagnosed diabetes and pre-diabetes. It has been widely accepted and used locally.

## Competing interests

Nothing to declare.

### Acknowledgements

We owe our sincere thanks to the local research teams from Qingdao Municipal Health Bureau and Qingdao Centers for Disease Control and Prevention, Qingdao, China for their contribution to the field survey (http://www.qddiabetes.org/Organize-6.asp). We would also like to thank the Finnish Academy (118492) for supporting the data analysis; the Bayer Healthcare in China and Lifescan of the Johnson & Johnson Company in China for providing support in the field surveys.

## References

1 Baan CA, Ruige JB, Stolk RP, Witteman JC, Dekker JM, Heine RJ *et al.* Performance of a predictive model to identify undiagnosed diabetes in a health care setting. *Diabetes Care* 1999; **22**: 213–219.
2 Griffin SJ, Little PS, Hales CN, Kinmonth AL, Wareham NJ. Diabetes risk score: towards earlier detection of type 2 diabetes in general practice. *Diabetes Metab Res Rev* 2000; **16**: 164–171.
3 American Diabetes Association. Screening for type 2 diabetes. *Diabetes Care* 2000; **23**: S20–S23.
4 Lindstrom J, Tuomilehto J. The diabetes risk score: a practical tool to predict type 2 diabetes risk. *Diabetes Care* 2003; **26**: 725–731.
5 Glumer C, Carstensen B, Sandbaek A, Lauritzen T, Jorgensen T, Borch-Johnsen K. A Danish diabetes risk score for targeted screening: The Inter99 study. *Diabetes Care* 2004; **27**: 727–733.


© 2010 The Authors.
Journal compilation © 2010 Diabetes UK. 

6 Mohan V, Deepa R, Deepa M, Somannavar S, Datta M. A simplified Indian diabetes risk score for screening for undiagnosed diabetic subjects. *J Assoc Physicians India* 2005; **53**: 759–763.
7 Ramachandran A, Snehalatha C, Vijay V, Wareham NJ, Colagiuri S. Derivation and validation of diabetes risk score for urban Asian Indians. *Diabetes Res Clin Pract* 2005; **70**: 63–70.
8 Aekplakorn W, Bunnag P, Woodward M, Sritara P, Cheepudomwit S, Yamwong S *et al*. A risk score for predicting incident diabetes in the Thai population. *Diabetes Care* 2006; **29**: 1872–1877.
9 Al-Lawati JA, Tuomilehto J. Diabetes risk score in Oman: a tool to identify prevalent type 2 diabetes among Arabs of the Middle East. *Diabetes Res Clin Pract* 2007; **77**: 438–444.
10 Schulze MB, Hoffmann K, Boeing H, Linseisen J, Rohrmann S, Mohlig M *et al*. An accurate risk score based on anthropometric, dietary, and lifestyle factors to predict the development of type 2 diabetes. *Diabetes Care* 2007; **30**: 510–515.
11 Balkau B, Lange C, Fezeu L, Tichet J, de Lauzon-Guillain B, Czernichow S *et al*. Predicting diabetes: clinical, biological, and genetic approaches: data from the Epidemiological Study on the Insulin Resistance Syndrome (DESIR). *Diabetes Care* 2008; **31**: 2056–2061.
12 Bindraban NR, van Valkengoed IG, Mairuhu G, Holleman F, Hoekstra JB, Michels BP *et al*. Prevalence of diabetes mellitus and the performance of a risk score among Hindustani Surinamese, African Surinamese and ethnic Dutch: a cross-sectional population-based study. *BMC Public Health* 2008; **8**: 271.
13 Cabrera de Leon A, Coello SD, Rodriguez Perez Mdel C, Medina MB, Almeida Gonzalez D, Diaz BB *et al*. A simple clinical score for type 2 diabetes mellitus screening in the Canary Islands. *Diabetes Res Clin Pract* 2008; **80**: 128–133.
14 Hippisley-Cox J, Coupland C, Robson J, Sheikh A, Brindle P. Predicting risk of type 2 diabetes in England and Wales: prospective derivation and validation of QDScore. *Br Med J* 2009; **338**: b880.
15 Gao W, Dong Y, Nan H, Tuomilehto J, Qiao Q. The likelihood of diabetes based on the proposed definitions for impaired fasting glucose. *Diabetes Res Clin Pract* 2008; **79**: 151–155.
16 WHO/IDF Consultation. *Definition and Diagnosis of Diabetes Mellitus and Intermediate Hyperglycemia: Report of a WHO/International Diabetes Federation Consultation*. Geneva: the World Health Organization Document Production Services, 2006.
17 Pencina MJ, D'Agostino RB Sr, D'Agostino RB Jr, Vasan RS Evaluating the added predictive ability of a new marker: from area under the ROC curve to reclassification and beyond. *Stat Med* 2008;7:157–172; discussion 207–212.
18 Gu D, Reynolds K, Duan X, Xin X, Chen J, Wu X *et al*. Prevalence of diabetes and impaired fasting glucose in the Chinese adult population: International Collaborative Study of Cardiovascular Disease in Asia (InterASIA). *Diabetologia*. 2003; **46**: 1190–1198.
19 Dong Y, Gao W, Nan H, Yu H, Li F, Duan W *et al*. Prevalence of Type 2 diabetes in urban and rural Chinese populations in Qingdao, China. *Diabet Med* 2005; **22**: 1427–1433.
20 Jia WP, Pang C, Chen L, Bao YQ, Lu JX, Lu HJ *et al*. Epidemiological characteristics of diabetes mellitus and impaired glucose regulation in a Chinese adult population: the Shanghai Diabetes Studies, a cross-sectional 3-year follow-up study in Shanghai urban communities. *Diabetologia* 2007; **50**: 286–292.
21 Saudek CD, Herman WH, Sacks DB, Bergenstal RM, Edelman D, Davidson MB. A new look at screening and diagnosing diabetes mellitus. *J Clin Endocrinol Metab* 2008; **93**: 2447–2453.
22 The International Expert Committee. International Expert Committee report on the role of the $A_{1c}$ assay in the diagnosis of diabetes. *Diabetes Care* 2009; **32**: 1327–1334.

© 2010 The Authors.
